# A MEMS-Based Flow Rate and Flow Direction Sensing Platform with Integrated Temperature Compensation Scheme

**DOI:** 10.3390/s90705460

**Published:** 2009-07-09

**Authors:** Rong-Hua Ma, Dung-An Wang, Tzu-Han Hsueh, Chia-Yen Lee

**Affiliations:** 1 Department of Mechanical Engineering, Chinese Military Academy, Kaohsiung 830, Taiwan; E-Mail: rh.ma@msa.hinet.net; 2 Institute of Precision Engineering, National Chung Hsing University, Taichung 402, Taiwan; E-Mail: daw@dragon.nchu.edu.tw; 3 Department of Mechanical and Automation Engineering, Da-Yeh University, Changhua 515, Taiwan; E-Mail: h18771646@yahoo.com.tw; 4 Department of Materials Engineering, National Pingtung University of Science and Technology, Pingtung 912, Taiwan

**Keywords:** cantilever, flow direction, flow sensor, MEMS, temperature compensation

## Abstract

This study develops a MEMS-based low-cost sensing platform for sensing gas flow rate and flow direction comprising four silicon nitride cantilever beams arranged in a cross-form configuration, a circular hot-wire flow meter suspended on a silicon nitride membrane, and an integrated resistive temperature detector (RTD). In the proposed device, the flow rate is inversely derived from the change in the resistance signal of the flow meter when exposed to the sensed air stream. To compensate for the effects of the ambient temperature on the accuracy of the flow rate measurements, the output signal from the flow meter is compensated using the resistance signal generated by the RTD. As air travels over the surface of the cross-form cantilever structure, the upstream cantilevers are deflected in the downward direction, while the downstream cantilevers are deflected in the upward direction. The deflection of the cantilever beams causes a corresponding change in the resistive signals of the piezoresistors patterned on their upper surfaces. The amount by which each beam deflects depends on both the flow rate and the orientation of the beam relative to the direction of the gas flow. Thus, following an appropriate compensation by the temperature-corrected flow rate, the gas flow direction can be determined through a suitable manipulation of the output signals of the four piezoresistors. The experimental results have confirmed that the resulting variation in the output signals of the integrated sensors can be used to determine not only the ambient temperature and the velocity of the air flow, but also its direction relative to the sensor with an accuracy of ± 7.5° error.

## 1. Introduction

Flow measurement is an essential task in many fields, including environmental monitoring, process control, medical instrumentation, air conditioning systems, weather forecasting systems, and so forth. For many years, flow sensing was accomplished using large-scale mechanical flow meters such as anemometers, turbines, Pitot tubes and so forth. However, in the mid-1980’s, Tai *et al.* [1] demonstrated the use of emerging micro-electro-mechanical systems (MEMS) techniques to fabricate a miniature anemometer based upon a polysilicon micro-bridge. In general, MEMS-based flow sensors have a smaller size, a greater sensitivity, a faster response and a lower power consumption than their traditional counterparts. Moreover, they are easily integrated with other IC devices and can be mass produced at a low cost. As a result, the literature contains a wealth of proposals for MEMS-based sensors for a diverse range of flow sensing applications [1–22].

Broadly speaking, these sensors can be categorized as either thermal or non-thermal, depending upon their mode of operation. Thermally-actuated gas flow sensors generally utilize some form of resistor arrangement to evaluate local temperature changes [2–11]. In such devices, the temperature differential between different resistors within the sensor varies as the gas flows through (or over) the sensor and can therefore be used to estimate the gas flow rate. For example, Neda *et al.* [2] proposed a single-wire-type thermal flow sensor with a polysilicon micro-heater capable of measuring gas flows with velocities ranging from 0.005∼35 ms^−1^. Lai *et al.* [3] presented an integrated flow sensor incorporating a silicon spreading-resistance temperature-sensing element, in which the flow velocity was automatically determined from the value of the heat transferred to the flowing fluid by a heated chip. Mailly *et al*. [4] used silicon micromachining techniques to fabricate an anemometer comprising a thin platinum (Pt) resistor deposited on a SiN*_x_*/Si substrate. The experimental results showed that the sensing performance was insensitive to both the ambient temperature and the direction of the gas flow due to its square layout of Pt resistor and high working temperature. Bruschi *et al*. [5] developed a gas flow sensor comprising two central heaters and two thermopiles located upstream and downstream of the heaters, respectively. When exposed to a gas flow, a flow-rate dependent temperature difference was generated between the two thermopiles, and it was shown that the magnitude of the flow rate could be inversely derived from the power mismatch between the two heaters when they were driven in such a way that the temperature gradient was canceled out. Buchner *et al.* [6] presented a high-temperature fabrication process for thermopile-based flow sensors. In the proposed approach, *p*-doped polysilicon and titanium-tungsten thermopiles were deposited on LPCVD silicon nitride membranes, which were then released using a DRIE process. It was shown that the high-temperature passivation of the silicon nitride layer reduced the tendency toward pinhole formation and ensured a good coverage of the underlying silicon substrate, thereby improving both the sensitivity and the response time of the sensor.

In a recent study, Dijkstra *et al*. [7] developed a calorimetric miniaturized flow sensor featuring planar sensor structures integrated within microchannels with a semicircular surface, and showed that the device exhibited a linear response for water flow rates up to approximately 300 nL min^−1^. Meng *et al.* [8] presented a biocompatible Parylene thermal flow sensing array for application in the bioMEMS and micro-total analysis systems (μ-TAS) fields. The proposed array enabled the simultaneous detection of both the flow rate and the flow direction, and proved capable of detecting flow rates as low as 0.5 μL/min using a low overheat ratio, thereby minimizing the heat supplied to the sensed medium.

Kim *et al.* [9] proposed a circular-type thermal flow direction sensor consisting of a heater surrounded by four sensing components. In the proposed device, the direction of the air flow was derived by analyzing the relative changes in the output signals of the four sensing components induced by variations in the air flow direction. It was shown that since changes in the ambient temperature exerted an equivalent effect on each of the four sensing components due to their symmetrical configuration, an accurate indication of the gas flow direction could be obtained without the need for any thermal compensation mechanism. In a later study, Kim *et al.* [10] utilized a similar configuration to realize a circular-type thermal flow sensor capable of evaluating both the flow direction and the flow velocity by measuring the relative changes in the output signals of the four detectors and monitoring the changes in the power signal supplied to the heater unit, respectively. Fürjes *et al.* [11] developed a calorimetric device for gas velocity and flow direction sensing comprising four temperature-sensing resistors arranged symmetrically around a central filament heater. It was shown that the device had a power consumption of 2∼20 mW for operating temperatures in the range 100∼500 °C.

In recent years, non-thermal gas flow meters have attracted considerable interest due to their lower power consumption and easier integration with other micro-scale systems than thermal gas flow sensors. The literature contains many proposals for such sensors, including differential pressure (DP) flow sensors utilizing fiber Bragg gratings (FBG) [12], multi-sensor chips for flow rate sensing based on shear stress and pressure measurements [13], lift force flow sensors with integrated hot-chips [14], flow meters based on dual FBG sensors and a cross-correlation technique [15], electrokinetic flow meters [16], electromagnetic flow meters [17], and vortex flow meters based on a dual-wall DP method [18]. Su *et al.* [19] fabricated an ultra-thin micromachined silicon cantilever-based flow sensor with an integrated strain gauge at its root. The results showed that the device had a low power consumption, a simple fabrication procedure, and a minimum measurement limit of around 7.0 cms^−1^. Wang *et al.* [20] presented an air flow sensor based upon a single free-standing microcantilever structure, and showed that the device had a sensitivity of 0.0284 Ω/ms^−1^ and was capable of measuring gas flow rates as high as 45 ms^−1^. In 2008, Lee *et al.* [21] proposed a flow sensor for flow rate and direction measurement. The flow rate and direction sensing units were made of a set of micro-heater/sensing resistor on a membrane and four piezoresistive cantilevers, respectively. Though the sensing principles of airflow rate and direction were presented in their study, a sensing algorithm was not investigated in detail and furthermore, an RTD for temperature compensation was not developed in their study. Lee *et al.* [22] also proposed a micro-sensor for flow direction measurement by arranging eight cantilever structures on an octagonal platform. In their study, as air traveled across the sensor, it displaced the upstream beam downward and the downstream beam upward. By measuring the resistor signals of each of the cantilever beams, the micro-sensor was capable of measuring the flow direction of the air passing over the sensor. Though good simulated results were obtained in their study, experimental results were still wondered to confirm the sensor characterization.

This study develops a MEMS-based flow sensor capable of obtaining simultaneous measurements of both the flow rate and the flow direction. The principal components of the proposed device include: (1) a cross-form configuration of four free-standing cantilever beams for flow direction sensing; (2) a circular hot-wire flow meter supported on a thin low-stress silicon nitride membrane for flow rate sensing, and (3) a planar resistive temperature detector (RTD) for ambient temperature sensing. The detailed designs and operational principles of each of these three components are discussed in the following section.

## 2. Design and Operating Principles

**Figure 1.**
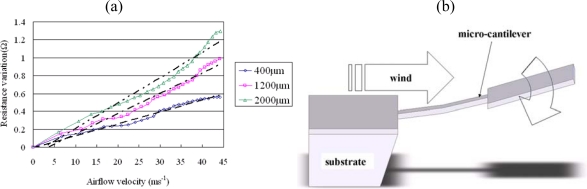
(a) Schematic illustration of micro-cantilever type flow sensor; (b) experimental (black lines) and theoretical (colored lines) results for flow rate sensitivity of sensors with different cantilever tip widths [20].

Kim *et al*. [10] presented a micromachined flow sensor for flow direction and velocity sensing applications in which four temperature sensing resistors were deposited symmetrically around a micro-heater on a silicon membrane. In the proposed approach, the flow direction was measured by analyzing the difference in the output signals of the four resistors as the air flowed over the surface of the membrane. The results showed that the sensor provided an accurate indication of the flow direction for air flows ranging from 0∼5 ms^−1^ and had a power consumption of approximately 80 mW. To reduce the power consumption and extend the operational range, Wang *et al*. [20] presented a MEMS-based flow sensor comprising a free-standing cantilever structure patterned with a platinum resistor [see Figure 1(a)]. The passage of an air flow over the surface of the cantilever caused the beam to deflect in the downward direction, and therefore induced a small increase in the resistance of the platinum resistor from which the flow rate could be inversely derived. It was shown that the device enabled the measurement of flow rates as high as 45 ms^−1^ and had a power consumption of just 0.02 mW. In their study, experimental and theoretical results showed good agreement and confirmed the feasibility of the cantilever type of flow sensor (Figure 1).

Incorporating the design concepts presented in References [10] and [20], the current study develops a MEMS-based flow sensor comprising a cross-form configuration of four free-standing cantilever beams. In the proposed approach, the gas flow direction is determined by comparing the output signals generated by four piezoresistors deposited on the upper surfaces of the cantilever structures. In addition, the flow rate is sensed by an integrated circular hot-wire flow meter. Finally, the ambient temperature is sensed using an RTD in order to compensate for the effects of changes in the local temperature on the flow rate measurements [10,11,20].

### 2.1. Gas Flow Direction Sensor

**Figure 2.**
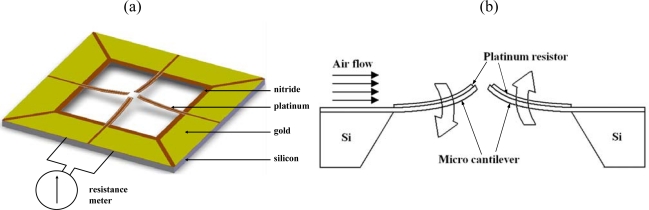
Schematic illustration showing deflection of microcantilevers as air flows over their surfaces. (a) Gas flow direction sensor comprising four microcantilevers arranged in cross-form configuration; (b) deflection of microcantilevers as air flows over the sensor surface.

As shown in Figure 2(a), the gas flow direction sensor has the form of four free-standing microcantilevers arranged in a cross-form configuration. Each cantilever has dimensions of 4,000 × 400 × 1 μm (length × width × thickness) and is patterned with a platinum piezoresistor with a length and width of 4,000 μm and 50 μm, respectively [23]. As air flows over the sensor, the cantilever beams in the upstream direction deflect in the downward direction, while those in the downstream direction deflect in the upward direction [see Figure 2(b)]. The deflection of the beams induces a change in the cross-sectional areas of the platinum resistors patterned on their upper surfaces and therefore produces a measurable change in their output signals. Since the amount by which each beam deflects is directly related to its orientation relative to the direction of the gas flow, the gas flow direction can be simply determined through an appropriate manipulation of the output signals of the four resistors.

### 2.2. Gas Flow Rate Sensor

For thermal-type flow meters, the heat created in the Pt resistor by the Joule heating effect is exchanged with the silicon substrate by conduction through the silicon nitride membrane or via convection or radiation through the air. To reduce the energy consumption and to increase the sensitivity of the device, it is therefore necessary to minimize the heat losses incurred by radiation and conduction. In general, the radiation losses are relatively small since the Pt resistor and silicon nitride layer have low emission coefficients. Meanwhile, the conduction losses can be minimized through the use of a suspended sensing structure. Consequently, the heat losses incurred by radiation and conduction, respectively, account for no more than 10% of the total heat losses [4]. In other words, the heat loss is dominated by convection and depends principally on the surface area of the suspended membrane. The power consumption (in Watts) of a thermal-type flow meter is given by:

(1) $$\begin{array}{l} P=\left(G_{\mathrm{cond}}+G_{\mathrm{conv}}+G_{\mathrm{rad}}\right)\left(T-T_{\mathrm{amb}}\right)\\ \approx G_{\mathrm{conv}}\left(T-T_{\mathrm{amb}}\right) \end{array}$$

where *G_cond_*, *G_conv_* and *G_rad_* (WK^−1^) are the thermal conductances associated with conduction, convection and radiation, respectively, and *T-T_amb_* (K) is the temperature rise. The thermal conductance associated with convection is given by:

(2) $$G_{\mathrm{conv}}={\mathrm{hS}}_{M}$$

where *h* (Wm^−2^K^−1^) is the coefficient of convection and *S_M_* (m^2^) is the total surface area of the two sides of the membrane. In performing flow rate measurements, the coefficient of convection, *h_CONV_*, is proportional to *v^n^*, where *v* is the flow velocity and *n* is an exponent which depends on the flow regime. In other words, *h* decreases as the flow rate and membrane temperature increase. In the present study, the change in the membrane temperature is measured using an RTD [4].

**Figure 3.**
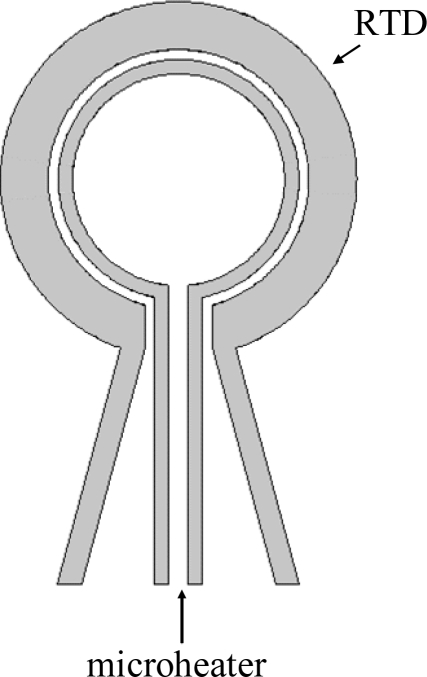
Schematic illustration of circular thermal flow meter.

As shown in Figure 3, the flow meter comprises a circular wire micro-heater (inner diameter: 4,400 μm; outer diameter: 5,000 μm) positioned within a circular RTD (inner diameter: 5,400 μm; outer diameter: 7,400 μm). Note that the micro-heater is positioned such that a constant gap of 200 μm is maintained between it and the circular RTD. During the sensing operation, an electrical voltage is supplied to the micro-heater, causing its temperature to rise. (Please note that a self-adjusted circuit of electrical voltage supply is connected with the micro-heater to keep the micro-heater temperature constant.) As a result, the output signal of the adjacent RTD also increases due to the heating effect of the radiated heat from the micro-heater. However, as any external flow reduces the total amount of heat transfer from the central circular heater to the outside circular temperature sensor, the upstream part of the circular temperature sensor is cooled down and the downstream part is heated up not much more than before. In practice, the amount of heat transferred from the micro-heater to the RTD varies inversely with the flow rate, and thus by measuring the change in the output signal of the RTD, the velocity of the gas flow can be inversely derived.

### 2.3. Ambient Temperature Detector

In a practical sensing application, the output signal from the circular RTD depends on both the heat transferred from the micro-heater (as governed by the flow rate) and the ambient temperature. Therefore, to obtain a reliable measurement of the gas flow rate, it is necessary to compensate for the ambient temperature effect. In the sensor developed in this study, this is accomplished by using the output signal of an unheated rectangular RTD with dimensions of 1,500 × 300 μm (length × width) to compensate the resistive signal of the thermal flow rate sensor.

## 3. Fabrication

**Figure 4.**
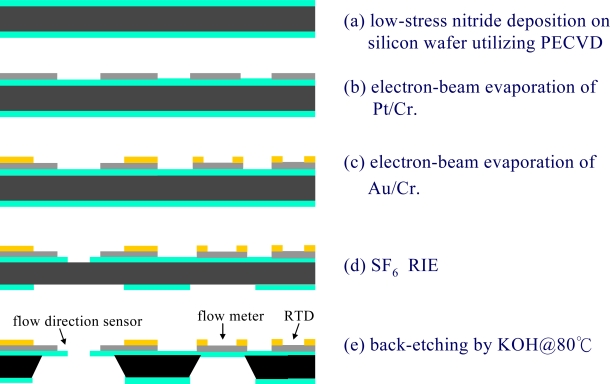
Schematic overview of fabrication process used to accomplish flow direction sensor with integrated flow meter and RTD.

Figure 4 presents a schematic illustration of the MEMS-based fabrication process used to realize the proposed integrated sensor platform. As shown, the fabrication process commenced by depositing a thin (1.0 μm) low-stress Si_3_N_4_ layer on either side of a double-side-polished silicon wafer utilizing a PECVD technique [see Figure 4(a)].

**Figure 5.**
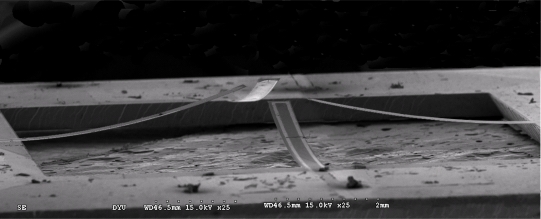
SEM image of flow direction sensor comprising four microcantilevers in a cross-form configuration.

Using an electron-beam evaporation process, a chromium (Cr) layer with a thickness of 0.03 μm was deposited on the upper Si_3_N_4_ surface to serve as an adhesion layer for the subsequent deposition of a Pt layer with a thickness of 0.1 μm. Platinum resistors for the flow direction sensor, flow velocity sensor and ambient temperature sensor were then patterned in a one-pass lithographic process [see Figure 4(b)]. The same deposition and patterning techniques were then re-applied to create thin (0.1 μm) gold (Au) lead electrodes on the end portions of each of the Pt resistors [see Figure 4(c)]. Finally, the cantilever/diaphragm structures and back-etching nitride mask were patterned in SF_6_ RIE plasma [see Figure 4(d)] and the freestanding structures were then released using a KOH etching agent (J.T Baker, 40 wt%, 80 °C) [see Figure 4(e)]. Figure 5 presents a scanning electron microscope (SEM) image of the cross-form flow direction sensor.

**Figure 6.**
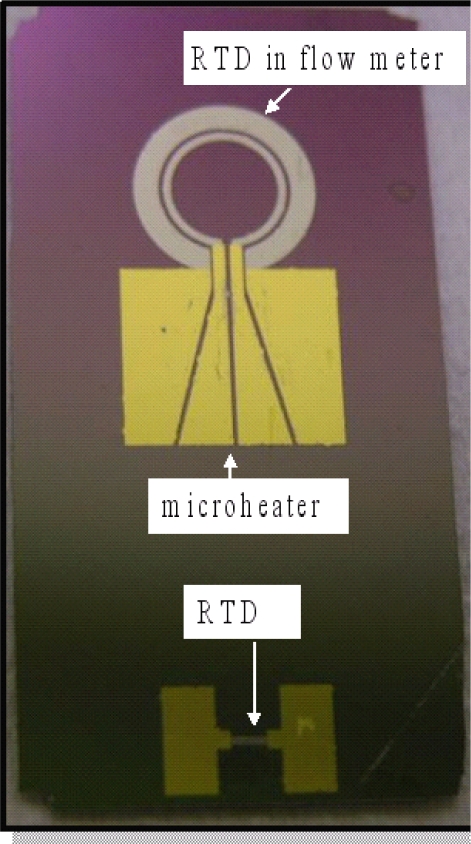
Photograph of circular thermal flow meter and RTD used for temperature compensation purposes (upper: flow meter, lower: RTD).

It is observed that each of the cantilever beams has a marked deflection in the upward direction as a result of the residual stress induced within the beam during the deposition and etching procedures in the fabrication process [23]. In practice, the curved characteristic of the cantilever beams is highly beneficial since it increases the tendency of the beams to deflect in either the upward or downward direction as air travels over the upper surface of the sensor, and therefore yields an improvement in the measurement sensitivity of the device. Figure 6 presents a photograph of the thermal flow sensor (comprising a circular micro-heater and RTD) and the rectangular RTD used to measure the ambient temperature.

## 4. Results and Discussion

**Figure 7.**
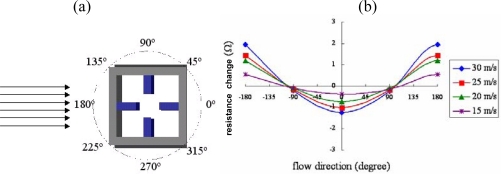
(a) Position of initial air flow direction relative to sensor; (b) correlation between resistance signal variations and air flow direction for air flow velocities in the range of 15∼30 ms^−1^.

The gas flow sensor was placed on a rotary table (LC-PR60, TanLian E-O Co., Ltd., Taiwan) and positioned in a wind tunnel such that the air passed over the sensor surface in the direction shown in Figure 7(a). As the sensor was rotated in the wind tunnel, the variation in the output resistance signal of the cross-form flow direction sensor was measured using a Load-Capacitance-Resistance (LCR) meter (Model 4263B, Agilent Technologies; working frequency 100∼100 KHz). The air flowed through the tunnel at velocities ranging from 15∼30 ms^−1^ (note that for reference purposes, the air flow velocity was also measured using a Pitot tube flow meter). The flow meter was characterized in the same wind tunnel at flow rates ranging from 0∼32 ms^−1^ and temperatures between 80 °C and 180 °C. Finally, the performance of the RTD used for temperature compensation purposes was evaluated at temperatures between 25 °C and 60 °C in a climate controlled test chamber (THS-A, KSON Instrument Technology Co., Taiwan).

### 4.1. Flow Direction Measurement

Flow direction characteristics were analyzed at every 45° interval when the wind speed was set to 15, 20, 25 and 30 ms^−1^. Figure 7 presents the corresponding results of the four piezoresistors located at 90° of adjacent layouts for different flow rate conditions. Figure 7(b) illustrates the variations of the output signals from the flow direction sensor as the sensor was rotated incrementally through 360° within the wind tunnel under gas flows rates of 15, 20, 25 and 30 ms^−1^, respectively. The results clearly show that a strong dependency exists between the resistance signal and the direction of the air flow relative to the sensor axes. Specifically, the signal obtains its maximum value when the air flow passes over the sensor surface with an orientation of ± 180°, but falls to a value close to zero at an orientation of ± 90°. Thus, it is inferred that the greatest contribution to the resistance signal is provided by the upstream and downstream piezoresistors, respectively, while that of the cantilever beams orientated perpendicular to the airflow direction is virtually zero. The results also show that the resistance signal of the flow direction sensor increases with an increasing flow rate due to the corresponding increase in the heat loss. In other words, extracting the true value of the flow direction from the sensor signal requires the flow rate to be known in advance. The experimental results presented in Figure 7 are in good agreement with those presented in References [10] and [11], and therefore confirm the feasibility of the proposed sensing platform for flow direction measurement applications.

### 4.2. Flow Rate Measurement

**Figure 8.**
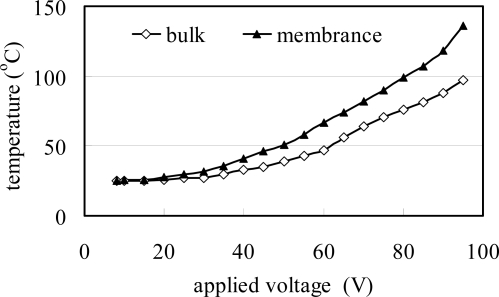
Variations of micro-heater temperature with magnitude of supplied electrical voltage for flow meters on diaphragm and bulk structure, respectively.

Figure 8 presents the variation of the circular micro-heater temperature with the magnitude of the applied voltage. Note that the figure presents results for both a membrane-mounted flow rate meter [see Figure 4(e)] and a bulk-mounted flow rate meter [see Figure 4(d)]. In both cases, it can be seen that the temperature increases with an increasing voltage. However, it is noted that for a given voltage, the temperature of the micro-heater suspended on the Si_3_N_4_ diaphragm is greater than that of the heater mounted on the bulk structure due to the higher convection effect caused by the lower thermal conduction in the suspended membrane.

**Figure 9.**
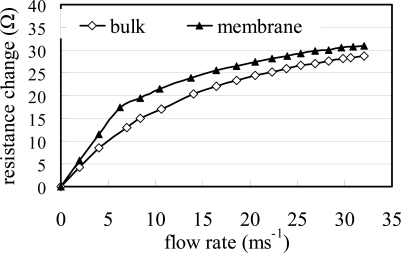
Variations of measured resistance change with flow rate for flow meters on diaphragm and bulk structure, respectively, at 100 °C where the initial resistance is 580.6 Ω.

**Figure 10.**
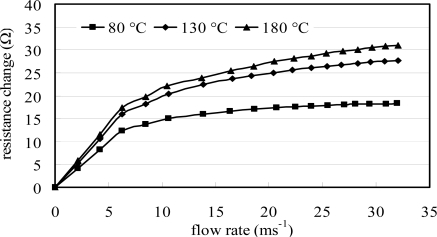
Variations of measured resistance change with flow rate for flow meter on diaphragm as function of micro-heater temperature where the initial resistances are 571.1 Ω, 594.8 Ω and 618.5 Ω, respectively (T = 80 °C, 130 °C, and 180 °C).

Figure 9 plots the resistance signals of the two flow meters against the flow rate for a constant micro-heater temperature of 100 °C. Again, it is observed that the sensitivity (i.e., the resistance change/the flow rate change) of the membrane-mounted flow meter is higher than that of the sensor mounted on the bulk structure. Therefore, the validity of the proposed membrane-mounted flow meter design is confirmed. Figure 10 shows the variation in the resistance signal of the suspended flow meter with the flow rate as a function of the micro-heater temperature (i.e., 80 °C, 130 °C and 180 °C, respectively). In general, it can be seen that the change in the resistance signal of the RTD in the flow meter increases as an approximately square-root function of the flow rate. The experimental results are in good agreement with both the predictions of Equations (1) and (2) and the findings presented in References [10] and [11]. Therefore the feasibility of the thermal flow meter for flow rate measurement applications is confirmed. In addition, it is observed that the sensitivity of the flow meter increases with an increasing micro-heater temperature due to the greater temperature change caused by the higher heat generation.

### 4.3. Temperature Measurement

**Figure 11.**
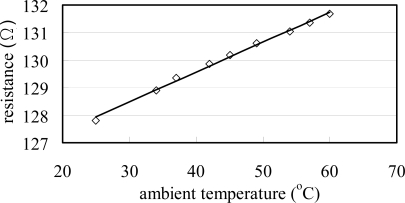
TCR plot of integrated RTD used for temperature compensation purposes (TCR = 0.00078 °C^−1^).

Figure 11 shows that the resistance signal of the rectangular RTD sensor increases linearly with the ambient temperature. Thus, the suitability of the RTD sensor for temperature compensation purposes is confirmed. From inspection, the average temperature coefficient of resistance (TCR) is found to be 0.00078 °C ^−1^.

### 4.4. Flow-Direction Evaluation

**Figure 12.**
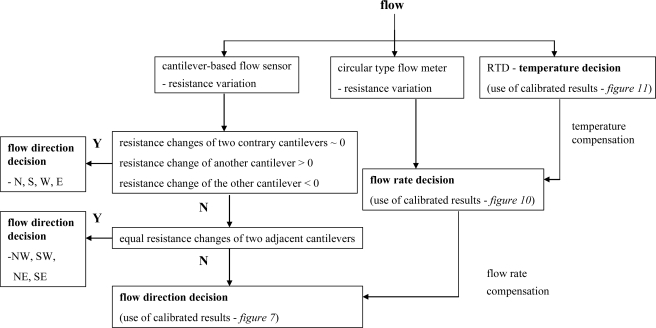
Flow chart of sensing platform operation.

Collectively, the three mechanisms described in Sections 4.1∼4.3 enable highly-precise simultaneous measurements of the air flow rate and the air flow direction to be obtained. As described in the following paragraph, in the proposed sensing platform, the air flow velocity is measured by the membrane-supported flow meter and is compensated with the signal obtained from the rectangular RTD, while the air flow direction is obtained from the cross-form cantilever sensor and is compensated with the temperature-calibrated flow rate obtained from the flow meter and integrated ambient temperature detector. The overall sensing operation is summarized in flow chart form in Figure 12.

As air flows over the surface of the sensor, a corresponding change is induced in the resistance signals obtained from the four piezoresistors in the flow direction sensor, the RTD element in the circular flow meter, and the RTD element in the ambient temperature detector, respectively. Utilizing the calibration curve presented in Figure 11, the temperature of the ambient surroundings is extracted from the resistance signal of the rectangular RTD sensor. Meanwhile, using the resistance signal obtained from the circular RTD, the flow rate is extracted from the calibration curves presented in Figure 10 in accordance with the temperature of the micro-heater (as derived from Figure 8 for the specified value of the applied voltage) appropriately compensated by the ambient temperature (as measured by the rectangular RTD sensor). Finally, the direction of the air flow is evaluated by analyzing the resistance signals obtained from the four piezoresistors patterned on the free-standing cantilevers. As shown in Figure 12, the air flow direction is evaluated using a three-step procedure. If the resistance signals of two opposing cantilevers exhibit no change, but the resistance signal of one of the remaining cantilevers increases in the positive direction while that of the other increases in the negative direction, the flow direction is assumed to be either N(orth), S(outh), W(est) or E(ast). Note that the particular flow direction is determined by inspecting which of the four cantilever beams exhibits the maximum resistance change in the positive direction (i.e., the upstream cantilever) and which beam exhibits the maximum resistance change in the negative direction (i.e., the downstream cantilever). In the event that the resistance signals of two adjacent piezoresistors exhibit a change of an approximately equal magnitude in an equivalent direction, while those of the other two piezoresistors also exhibit a change of an approximately equal magnitude but in the opposite direction, the direction of the air flow is assumed to bisect the angle between each pair of cantilevers, and thus the air flow direction is determined to be NW, SW, NE or SE. The actual flow direction is determined simply by identifying which set of resistors exhibits the maximum change in the positive direction and which set exhibits the maximum change in the negative direction. If the resistance signals satisfy neither of the two conditions described above, the flow direction is extracted from the calibration curves presented in Figure 7(b) in accordance with the temperature-compensated flow rate obtained from the flow rate sensor.

### 4.5. Measurement Accuracy of Flow Direction Sensor

**Figure 13.**
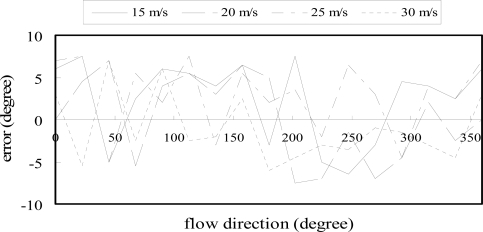
Measurement accuracy of flow-rate-compensated flow direction sensor at various values of the gas flow rate.

The measurement accuracy of the flow-rate-compensated flow direction sensor was evaluated by measuring the changes in the output signals of the four piezoresistors as the sensor was rotated incrementally from 0° to 360° under gas flow rates of 15 ms^−1^, 20 ms^−1^, 25 ms^−1^, and 30 ms^−1^, respectively, at an ambient temperature of 25 °C. Figure 13 illustrates the variation of the measurement accuracy with the orientation of the sensor relative to the air flow direction. Note that the measurement accuracy is defined here as the difference between the air flow direction derived from the temperature-compensated signal of the flow direction sensor and the actual orientation of the rotary table. From inspection, the measurement accuracy of the air flow direction sensor is found to be approximately ± 7.5°.

### 4.6. Sensing Hysteresis of the Cantilever-based Flow Sensor

**Figure 14.**
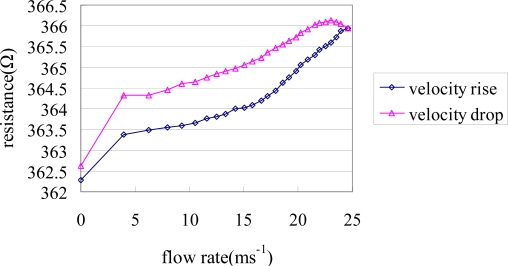
Sensing hysteresis of cantilever-based air flow sensor.

Ideally, a sensor should follow the same resistance path as the air flow rate is increased or decreased. However, in practice, most sensors exhibit a small degree of hysteresis. Figure 14 shows the sensing hysteresis of the individual cantilever-based flow sensor. In this investigation, the temperature was maintained constant at 25 °C while the air flow rate was progressively increased from 0 ms^−1^ to 25 ms^−1^ over a period of 20 min and then reduced to 0 ms^−1^ at the same rate. The results show that the cantilever-based flow sensor has a hysteresis of 20% F.S., which implies that sensing hysteresis is an important consideration in the design of cantilever-based sensors.

### 4.7. Stability of the Cantilever-Based Flow Sensor

The experimental results indicate that at a constant flow rate of 25 ms^−1^ and a temperature of 25 °C, the variations in stability of the cantilever-based flow sensor are small, i.e., 0.24% F.S. The total time of measurement period was 120 min.

## 5. Conclusions

This study has fabricated a MEMS-based micro-flow sensor comprising a flow direction sensor, a flow rate sensor, and an ambient temperature sensor. The flow direction sensor consists of a cross-form configuration of four free-standing cantilevers, each patterned with a pizeoresistor. The passage of air over the sensor surface prompts a deflection of the cantilevers, which in turn produces a measurable change in the output signals of the four resistors from which the direction of the air flow can then be derived. The flow rate sensor comprises a circular wire micro-heater positioned within a circular resistive temperature detector (RTD). When exposed to an air flow, the amount of heat transferred from the micro-heater to the RTD reduces and therefore causes a corresponding reduction in the output signal of the RTD from which the air flow rate can be inversely derived. Finally, the ambient temperature detector comprises a rectangular RTD whose output signal varies linearly with changes in the ambient temperature. In the sensing operation, the accuracy of the flow rate measurement is improved by compensating the resistance signal obtained from the circular RTD in accordance with the signal obtained from the ambient temperature detector. The dynamic response of the four cantilevers in the air flow direction sensor is dependent not only on the direction of the air flow, but also upon the flow velocity. Therefore, to ensure the precision of the air flow direction measurement, the output signal of the flow direction sensor is compensated using the temperature-corrected value of the air flow rate obtained from the circular flow rate sensor. Overall, the experimental results have confirmed that the proposed integrated micro-flow sensor is capable of measuring the direction of the sensed air flow with an accuracy of ± 7.5°. Consequently, the sensing platform provides an ideal solution for flow sensing applications in indoor and outdoor environments of living spaces.
